# P-941. Put a CAP on Antibiotics: Electronic Medical Record Tools Improve Antibiotic Prescribing at Discharge for Community Acquired Pneumonia

**DOI:** 10.1093/ofid/ofaf695.1144

**Published:** 2026-01-11

**Authors:** Merin Babu, Amy Beaulac, Janeen Dubay, Lori Leman, Anita Shallal, Erin Eriksson, Sairia Dass, Megan M Cahill, Rachel M Kenney, Brian Church, Robert McCollom, Abigail Geyer, Michael P Veve, Sage Greenlee

**Affiliations:** Henry Ford Health, Macomb, Michigan; Henry Ford West Bloomfield Hospital, West Bloomfield, Michigan; Henry Ford West Bloomfield Hospital, West Bloomfield, Michigan; Henry Ford Macomb Hospital, Macomb, Michigan; Henry Ford Hospital, Detroit, Michigan; Henry Ford Jackson Hospital, Jackson, Michigan; Henry Ford Jackson Hospital, Jackson, Michigan; Henry Ford Macomb Hospital, Macomb, Michigan; Henry Ford Hospital, Detroit, Michigan; Henry Ford Health, Macomb, Michigan; Henry Ford Health, Macomb, Michigan; Henry Ford Wyandotte Hospital, Wyandotte, Michigan; Eugene Applebaum College of Pharmacy and Health Sciences, Detroit, MI; Henry Ford Macomb Hospital, Macomb, Michigan

## Abstract

**Background:**

Guidelines recommend that uncomplicated community acquired pneumonia (CAP) is treated for 5 days; however, patients are commonly prescribed excessive antibiotics at discharge. This study evaluated the impact of electronic medical record (EMR) transitions of care (TOC) tools on duration of therapy for uncomplicated CAP.

**Figure 1.**
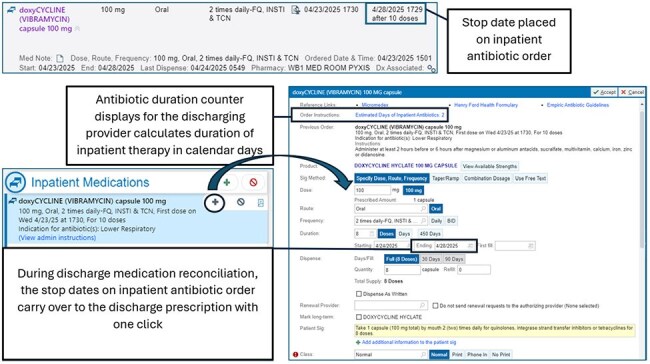
Transitions of Care EMR Tools

**Figure 2.**
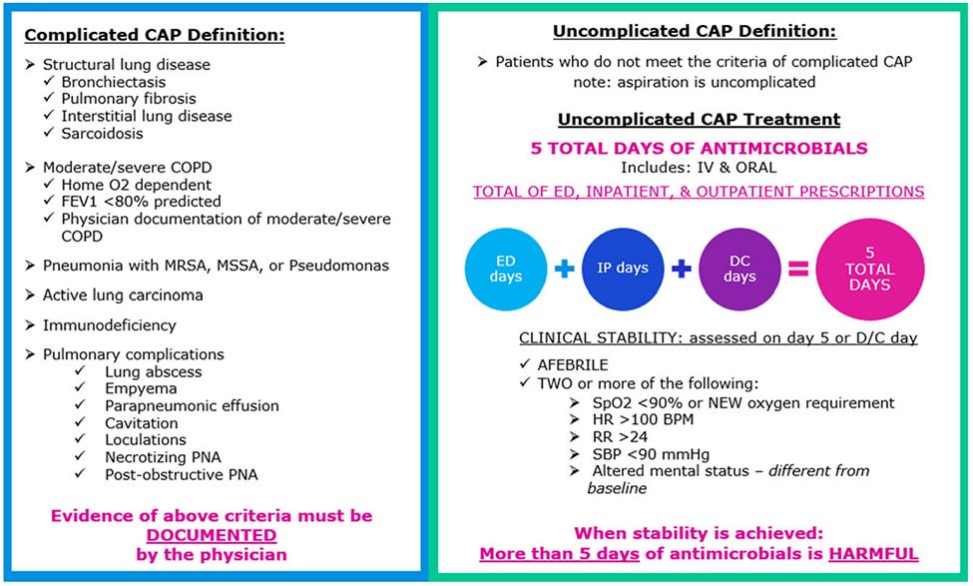
Complicated CAP

**Methods:**

IRB approved, single pre-, post-test quasi-experiment of hospitalized adult patients with uncomplicated CAP between 07/01/2023-11/30/2023 (pre-intervention) and 07/01/2024-11/30/2024 (post-intervention). EMR TOC tools implemented in March-May 2024, included total antibiotic days counter and inpatient stop date carry-over on discharge order (Figure 1). Patients who completed antibiotics prior to discharge date, admitted to intensive care unit, respiratory culture with methicillin-resistant *Staphylococcus aureus* or *Pseudomonas aeruginosa* ≤ 12-months before admission, suspected concomitant infection, or complicated CAP (Figure 2) were excluded. The primary outcome was the proportion of patients prescribed < 6-calendar-days of total therapy for CAP. Secondary outcomes included 30-day CAP-related readmission and *Clostridioides difficile* infection (CDI), multidrug-resistant organisms (MDRO) ≤ 90-days of discharge, and days of therapy prescribed at discharge.

**Table 1.**
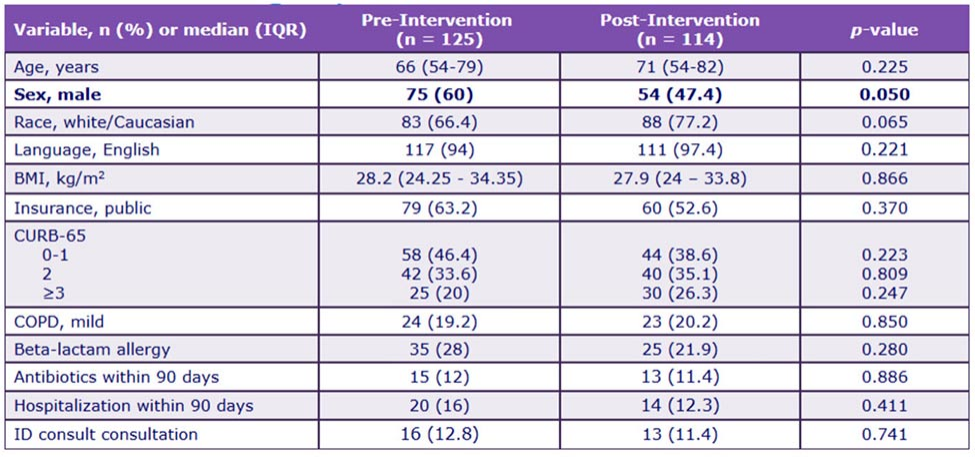
Patient Demographics

**Table 2.**
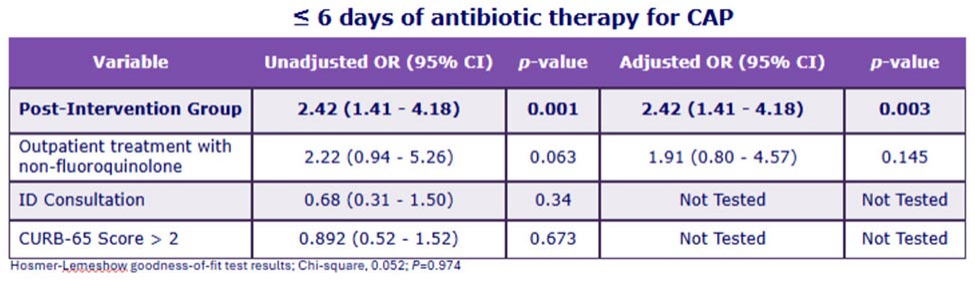
Regression Analysis

**Results:**

A total of 239 patients were included; 125 patients in the pre-intervention period and 114 patients in the post-intervention period. Demographics are noted in Table 1. A higher proportion of patients in the post-intervention group received < 6-days of CAP therapy compared to the pre-intervention group (53.6% pre- vs. 73.7% post-intervention, *P*=0.001). There were no differences in readmission, CDI, or MDRO infection between groups. Post-intervention group patients were prescribed shorter median (IQR) antibiotic duration at discharge than pre-intervention group patients (3 [2-4] pre- vs. 2.5 [1-4] post-intervention, *P*< 0.001). After adjustments for confounders, patients in the post-intervention group had 2-fold increased odds of receiving < 6-days of therapy for CAP (Table 2).

**Conclusion:**

Implementation of EMR TOC tools significantly improved optimal CAP prescribing in hospitalized adults, with no differences in patient outcomes.

**Disclosures:**

All Authors: No reported disclosures

